# Low Toe–Brachial Index Is Associated With Stroke Outcome Despite Normal Ankle–Brachial Index

**DOI:** 10.3389/fneur.2021.754258

**Published:** 2021-12-20

**Authors:** Minho Han, Young Dae Kim, Ilhyung Lee, Hyungwoo Lee, Joonnyung Heo, Hye Sun Lee, Hyo Suk Nam

**Affiliations:** ^1^Department of Neurology, Yonsei University College of Medicine, Seoul, South Korea; ^2^Integrative Research Center for Cerebrovascular and Cardiovascular Diseases, Yonsei University College of Medicine, Seoul, South Korea; ^3^Department of Research Affairs, Biostatistics Collaboration Unit, Yonsei University College of Medicine, Seoul, South Korea

**Keywords:** ankle-brachial index, peripheral artery disease, prognosis, stroke, toe-brachial index

## Abstract

**Introduction:** We investigated whether the toe–brachial index (TBI) is associated with stroke prognosis and evaluated this association in patients with normal ankle–brachial index (ABI).

**Methods:** Acute ischemic stroke patients who underwent TBI measurements were enrolled. Poor functional outcome was defined as modified Rankin Scale score ≥3. Major adverse cardiovascular event (MACE) was defined as stroke recurrence, myocardial infarction, or death. Normal ABI was defined as 0.9 ≤ ABI ≤ 1.4.

**Results:** A total of 1,697 patients were enrolled and followed up for a median 39.7 (interquartile range, 25.7–54.6) months. During the period, 305 patients suffered MACE (18.0%), including 171 (10.1%) stroke recurrences. TBI was associated with hypertension, diabetes, atrial fibrillation, aortic plaque score, ABI, and brachial–ankle pulse wave velocity (all *p* < 0.05). In multivariable logistic regression, TBI was inversely associated with poor functional outcome in all patients [odds ratio (OR) 0.294, 95% confidence interval (CI) 0.114–0.759], even in patients with normal ABI (OR 0.293, 95% CI 0.095–0.906). In multivariable Cox regression, TBI < 0.6 was associated with stroke recurrence [hazard ratio (HR) 1.651, 95% CI 1.135–2.400], all-cause mortality (HR 2.105, 95% CI 1.343–3.298), and MACE (HR 1.838, 95% CI 1.396–2.419) in all patients. TBI < 0.6 was also associated with stroke recurrence (HR 1.681, 95% CI 1.080–2.618), all-cause mortality (HR 2.075, 95% CI 1.180–3.651), and MACE (HR 1.619, 95% CI 1.149–2.281) in patients with normal ABI.

**Conclusions:** Low TBI is independently associated with poor short- and long-term outcomes in acute ischemic stroke patients despite normal ABI.

## Introduction

Peripheral arterial disease (PAD) is significantly associated with future ischemic stroke and vascular events (1). The low ankle–brachial index (ABI < 0.9) is the most common and well-established marker for diagnosing PAD (2). Our previous studies demonstrated that low ABI is closely associated with initial stroke severity (3) and poor functional outcome in patients with acute ischemic stroke (4). Additionally, low ABI has been reported as a predictor of stroke recurrence and mortality (5). However, ABI is sometimes inaccurate because of peripheral artery calcification. Medial artery calcification is a non-obstructive calcification of the tunica media that commonly occurs in lower limb arteries, leading to elevated ABI values (6). Patients with stroke are often elderly and have diabetes and chronic kidney disease. These risk factors are associated with medial artery calcification (7, 8). Therefore, using ABI alone to identify PAD may misdiagnose PAD in patients with stroke.

The toe–brachial index (TBI) may be the alternative to overcome the limitation of ABI (9). TBI, defined as the ratio of the systolic blood pressure in the great toe to the brachial artery, is more sensitive to PAD detection in patients with medial artery calcification because smaller, more distal arteries are not vulnerable to calcification (10). Thus, the American Heart Association (2, 11) and the American Diabetic Association (12) have recommended measuring TBI in patients with suspected medial artery calcification. To the best of our knowledge, no studies have evaluated the prognostic value of TBI in patients with ischemic stroke. Particularly, the association between TBI and the prognosis of patients with normal ABI has not been reported. Therefore, we hypothesized and investigated whether low TBI is associated with poor functional outcome, stroke recurrence, all-cause mortality, and major adverse cardiovascular event (MACE) in patients with acute ischemic stroke. We further evaluated whether the associations between TBI and stroke prognosis are valid in patients with normal ABI.

## Materials and Methods

### Study Population

This is a hospital-based observational study on patients with ischemic stroke who were prospectively registered to a stroke registry from January 2012 to December 2018. The registry enrolled consecutive patients with acute ischemic stroke within 7 days of onset. During admission, all patients were tested for TBI with ABI measurements and evaluated with brain MRI and/or CT, as well as cerebral angiography (magnetic resonance angiography, CT angiography, or digital subtraction angiography). Systemic evaluation included 12-lead electrocardiography, chest radiography, standard blood tests, and lipid profile during admission in the stroke unit. To determine the cardioembolic source and aortic atherosclerosis, all study patients were thoroughly evaluated *via* transesophageal echocardiography (TEE) and continuous electrocardiography monitoring. TEE was conducted within 2 weeks of the initial stroke. TEE was a part of the standard evaluation for all patients, except in those with decreased consciousness, impending brain herniation, poor systemic conditions, tracheal intubations, or failure of introducing an esophageal transducer. A patient was also excluded from undergoing TEE if informed consent for TEE was not obtained from either the patient or family members (13, 14). Patients were treated using standard treatment protocols based on the guidelines for acute ischemic stroke (15, 16). Stroke classification was determined during weekly conferences. Based on the consensus of three stroke neurologists, stroke subtypes were classified according to the Trial of ORG 10,172 in Acute Stroke Treatment classification (17). The Institutional Review Board of Severance Hospital, Yonsei University Health System, approved this study and waived the need for informed consent because of the retrospective design and observational nature of this study (IRB: 4-2021-0023).

### Clinical Variables

We collected data on baseline characteristics [age, sex, and National Institutes of Health Stroke Scale (NIHSS) score for neurological deficit] upon admission, presence of risk factors, and laboratory data (glucose, high-density lipoprotein, and low-density lipoprotein). Hypertension was defined as systolic blood pressure ≥140 mmHg or diastolic blood pressure ≥90 mmHg after repeated measurements during hospitalization or currently taking antihypertensive medication. Diabetes was defined as fasting plasma glucose levels ≥126 mg/dL or taking an oral hypoglycemic agent or insulin. Hypercholesterolemia was defined as serum total cholesterol ≥240 mg/dL, low-density lipoprotein ≥160 mg/dL, or any history of use of lipid-lowering agents after a diagnosis of hypercholesterolemia. Current smoking was defined as having smoked a cigarette within 1 year before admission. Coronary artery disease was diagnosed when patients had a previous history (acute myocardial infarction, unstable angina, coronary artery bypass graft, or percutaneous coronary artery stent/angioplasty) or the presence of significant stenosis (≥50%) in any of the three main coronary arteries.

### Vascular Evaluations

Aortic plaques were identified *via* TEE and classified as complex or simple aortic plaques. Complex aortic plaques consisted of plaques protruding into the lumen by ≥4 mm and mobile lesions located in the proximal aorta. Simple aortic plaques were plaques <4 mm in the proximal aorta or plaques of any size located in the descending aorta (13, 14). The total aortic plaque score was defined as the sum of the number of complex and simple aortic plaques.

The TBI, ABI, and brachial–ankle pulse wave velocity (baPWV) were measured in the supine position using an automatic device (VP-1,000; Colin Co., Ltd., Komaki, Ja-pan), which has been validated previously (18). The TBI was calculated as the ratio of the systolic blood pressure of the great toe divided by the higher systolic blood pressure of the arms. The lower TBI values of both sides were used for the analysis. The cutoff value of TBI was set to < 0.6 based on a previous study (9), showing a higher kappa value than TBI < 0.7 for ABI < 0.9 (*k* = 0.369 vs. 0.187, all *p* < 0.001). The ABI was calculated as the ratio of the ankle systolic blood pressure divided by the higher systolic blood pressure of the arms. The lower ABI values of both sides were used for the analysis. Patients with low ABI were defined as having ABI < 0.9 on either side (19). Lower limb calcification is usually defined as ABI > 1.4; thus, patients with high ABI were defined as having ABI > 1.4 on either side (2, 19). Therefore, normal ABI was defined as 0.9 ≤ ABI ≤ 1.4. The baPWV on each side was automatically calculated as the transmission distance divided by the transmission time and expressed in centimeters per-second. Transmission distance from the arm to each ankle was automatically calculated according to the patient's height. Transmission time was defined as the time interval between the initial increase of brachial and tibial waveforms. The average of both baPWV was used for the analysis. The average of limb systolic and diastolic blood pressure was also used for the analysis. TBI and ABI measurements were performed by three investigators. Intra-rater reliability for TBI and ABI parameters was good to excellent as follows: right TBI [intraclass correlation coefficient (ICC) = 0.939, *p* < 0.001], left TBI (ICC = 0.978, *p* < 0.001), right ABI (ICC = 0.983, *p* < 0.001), and left ABI (ICC = 0.987, *p* < 0.001). Inter-rater reliability for TBI and ABI parameters was also good to excellent as follows: right TBI (ICC = 0.961, *p* < 0.001), left TBI (ICC = 0.919, *p* < 0.001), right ABI (ICC = 0.945, *p* < 0.001), and left ABI (ICC = 0.926, *p* < 0.001). TBI and ABI measurements were usually performed at one time. We repeated tests in patients with ABI < 1.0, interarm or interankle blood pressure difference ≥ 10 mmHg, or TBI ≤ 0.6 for reliability.

### Follow-Up and Outcomes

Patients were followed up in the outpatient clinic or by structured telephone interview at 3 months and yearly after discharge. Short-term functional outcomes at 3 months were determined *via* a structured interview using the modified Rankin Scale (mRS). Poor functional outcome was defined as mRS ≥ 3. Stroke recurrence was defined as newly developed neurologic symptoms with relevant lesions on brain CT and/or MRI 7 days after an index stroke or hospital discharge. MACE was defined as stroke recurrence, myocardial infarction occurrence, or death. The censoring date was December 31, 2019.

### Statistical Analysis

Software packages such as SPSS (version 25, SPSS, Chicago, IL, United States) and R package (version 4.0.5, http://www.R-project.org) were used for the statistical analysis. Intergroup statistical analyses were conducted to compare the demographic characteristics and risk factors in all study patients. The statistical significance of intergroup differences was assessed using the chi-squared test for categorical variables and independent two-sample *t*-test or Mann–Whitney U-test for continuous variables. Data are expressed as means ± SD or median (interquartile range) for continuous variables and number (%) for categorical variables. The chi-squared test for trend was conducted to investigate the association between TBI < 0.6 and the number of risk factors. The correlations between TBI and vascular markers were determined using Pearson's correlation analysis. Multivariable logistic regression analysis was conducted after adjusting for age, sex, initial stroke severity, and variables with *p* < 0.05 in univariate analysis to investigate the association between TBI and short-term functional outcome. Survival curves were generated according to the Kaplan–Meier method and compared using the log-rank test. Multivariable Cox proportional hazard regression was conducted to investigate the independent association between TBI and long-term outcomes. Harrell's C-index was used to assess whether low TBI improves predictive models of long-term outcomes. All *p*-values were two-tailed, and differences were considered significant at *p* < 0.05.

## Results

### Demographic Characteristics

A total of 2,449 consecutive ischemic stroke patients were enrolled during the study period. After the exclusion of 752 patients without TBI measurements, 1,697 patients were included for further analysis. Table 1 summarizes the baseline characteristics of the study population. The mean age of all study patients was 64.6 ± 13.5 years, and 1,033 (60.9%) patients were men. The median NIHSS score at admission was 2.0 (interquartile range, 1.0–5.0) (Table 1).

**Table 1:** Patient demographic and clinical characteristics.

	**Total**	**Good outcomes**	**Poor outcomes**	***p*-value**
		**(mRS of 0–2;**	**(mRS of 3–6;**	
	**(*n* = 1,697)**	***n* = 1,302)**	***n* = 395)**	
Age, year	64.6 ± 13.5	63.5 ± 13.5	67.9 ± 12.9	<0.001
Men	1,033 (60.9)	816 (62.7)	217 (54.9)	0.006
NIHSS score at admission	2.0 [1.0, 5.0]	2.0 [0.1, 3.0]	5.0 [3.0, 10.0]	<0.001
**Risk factors**
Hypertension	1,268 (74.7)	946 (72.7)	322 (81.5)	<0.001
Diabetes mellitus	530 (31.2)	378 (29.0)	152 (38.5)	<0.001
Hypercholesterolemia	361 (21.3)	272 (20.9)	89 (22.5)	0.485
Current smoking	368 (21.7)	299 (23.0)	69 (17.5)	0.02
Coronary artery disease	585 (34.5)	462 (35.5)	123 (31.1)	0.112
Atrial fibrillation	306 (18.0)	225 (17.3)	81 (20.5)	0.144
**Stroke subtype**
CE	474 (27.9)	358 (27.5)	116 (29.4)	0.116
LAA	265 (15.6)	189 (14.5)	76 (19.2)	
SVO	142 (8.4)	114 (8.8)	28 (7.1)	
SUD	761 (44.8)	599 (46.0)	162 (41.0)	
SOD	55 (3.2)	42 (3.2)	13 (3.3)	
**Laboratory findings**
Glucose, mg/dL	117.1 ± 56.3	114.5 ± 59.8	125.4 ± 42.1	0.001
HDL, mg/dL	44.5 ± 15.5	44.4 ± 11.5	44.9 ± 24.4	0.519
LDL, mg/dL	102.8 ± 35.9	103.0 ± 35.5	101.9 ± 37.2	0.562
**Vascular markers**
Complex aortic plaque score	1.8 ± 1.4	1.8 ± 1.4	1.9 ± 1.3	0.03
Simple aortic plaque score	2.8 ± 1.8	2.7 ± 1.8	3.1 ± 1.7	0.002
Total aortic plaque score	4.6 ± 3.0	4.5 ± 3.0	5.0 ± 2.8	0.004
baPWV, cm/s	1961.3 ± 584.5	1898.1 ± 533.2	2169.7 ± 689.3	<0.001
**ABI and TBI measurements**
Heart rate, bpm	71.0 ± 14.8	70.1 ± 14.0	74.3 ± 17.0	<0.001
Brachial SBP, mmHg	145.5 ± 22.2	144.9 ± 22.0	147.5 ± 23.0	0.051
Brachial DBP, mmHg	82.5 ± 17.0	82.6 ± 17.9	82.1 ± 14.0	0.604
Ankle SBP, mmHg	163.9 ± 28.0	163.6 ± 27.4	164.9 ± 30.1	0.463
Ankle DBP, mmHg	80.6 ± 14.8	80.4 ± 14.1	81.2 ± 16.9	0.423
Toe SBP, mmHg	107.9 ± 24.3	108.8 ± 23.5	105.1 ± 26.5	0.013
ABI <0.9	132 (7.8)	96 (7.4)	36 (9.1)	0.258
ABI > 1.4	31 (1.8)	13 (1.0)	18 (4.6)	<0.001
0.9 ≤ ABI ≤ 1.4	1,534 (90.4)	1,193 (91.6)	341 (86.3)	0.002
TBI	0.71 ± 0.15	0.72 ± 0.14	0.67 ± 0.16	<0.001
TBI <0.6	311 (18.3)	208 (16.0)	103 (26.1)	<0.001

*Data are expressed as means ± SDs, medians [interquartile ranges], or numbers (%). ABI, ankle-brachial index; baPWV, brachial-ankle pulse wave velocity; CE, cardioembolism; DBP, diastolic blood pressure, HDL, high-density lipoprotein; LAA, large artery atherosclerosis; LDL, low-density lipoprotein; mRS, modified Rankin Scale; NIHSS, National Institutes of Health Stroke Scale; SBP, systolic blood pressure; SOD, stroke of other determined cause; SVO, small vessel occlusion; SUD, stroke of undetermined cause; TBI, toe-brachial index*.

### Factors Associated With TBI

**Figure 1:**
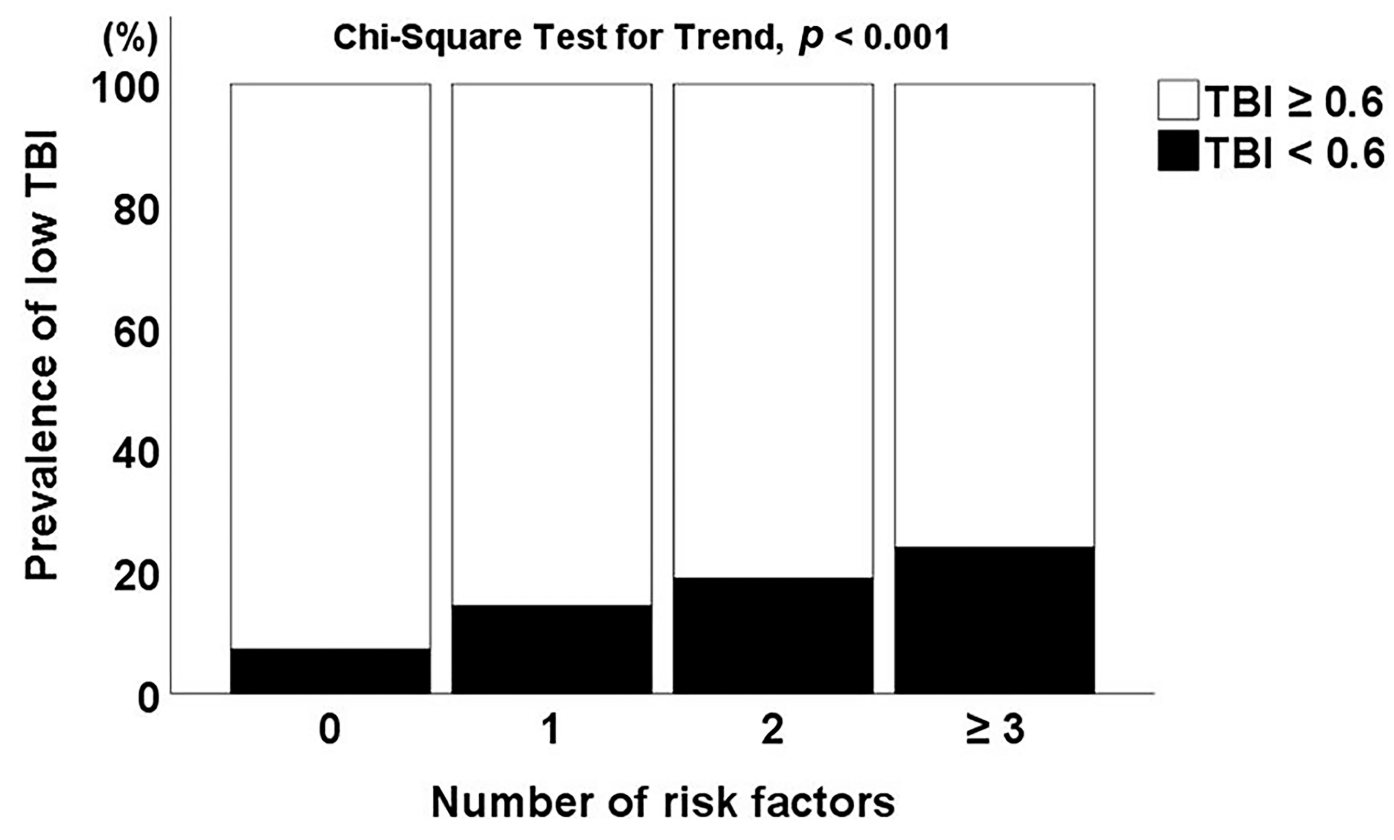
Correlation between low TBI and number of risk factors. TBI, toe-brachial index.

In all study patients, 18.3% patients showed low TBI (TBI <0.6). Among risk factors, TBI < 0.6 was associated with hypertension and diabetes (all *p* < 0.05). Atrial fibrillation was more frequently found in patients with TBI < 0.6 (25.7%) compared with TBI ≥ 0.6 patients (16.3%) (*p* < 0.001) (Supplementary Table 1). The presence of TBI < 0.6 was increased as the number of risk factors increased (*p* < 0.001 for trend) (Figure 1). For central and peripheral atherosclerotic markers, a continuous variable of TBI was negatively correlated with complex (*r* = −0.220), simple (*r* = −0.184), and total aortic plaque scores (*r* = −0.210) (all *p* < 0.001). TBI had a negative correlation with baPWV (*r* = −0.115) and a positive correlation with ABI (*r* = 0.530) (all *p* < 0.001) (Supplementary Table 2).

### Poor Functional Outcomes

Poor functional outcome at 3 months was observed in 395 (23.3%) patients. In univariable analysis, the poor functional outcome at 3 months was associated with old age, female sex, initial stroke severity, hypertension, diabetes, non-smoker, high glucose level, aortic plaque scores (complex, simple, and total), heart rate, baPWV, ABI > 1.4, 0.9 ≤ ABI ≤ 1.4, and lower TBI (continuous TBI value and TBI < 0.6 cutoff) (all *p* < 0.05) (Table 1). Multivariable logistic regression analysis showed that a continuous variable of TBI was independently associated with poor functional outcome at 3 months [odds ratio (OR) 0.294, 95% confidence interval (CI) 0.114–0.759; *p* = 0.011] in all patients (*n* = 1,697). A continuous variable of TBI was also associated with poor functional outcome (OR 0.293, 95% CI 0.095–0.906; *p* = 0.033) in patients with normal ABI (*n* = 1,534) (Table 2). However, the TBI < 0.6 cutoff was not significant in all study patients and normal patients with ABI.

**Table 2:** Logistic regression analysis of TBI for poor functional outcomes at 3 months.

	**Univariable**		**Multivariable^a^**	
	**OR (95% CI)**	***p*-value**	**OR (95% CI)**	***p*-value**
**All patients (*****n*** **=** **1,697)**				
TBI	0.097 (0.045–0.210)	<0.001	0.294 (0.114–0.759)	0.011
TBI <0.6	1.855 (1.418–2.428)	<0.001	1.342 (0.968–1.863)	0.078
**Patients with normal ABI (*****n*** **=** **1,534)**				
TBI	0.081 (0.032–0.203)	<0.001	0.293 (0.095–0.906)	0.033
TBI <0.6	1.732 (1.251–2.400)	0.001	1.241 (0.835–1.845)	0.285

*Data were derived from univariable and multivariable logistic regression analyses. ABI, ankle-brachial index; CI, confidence interval; NIHSS, National Institutes of Health Stroke Scale; OR, odds ratio; TBI, toe-brachial index*.

a *adjusted for age, sex, NIHSS score at admission, hypertension, diabetes mellitus, current smoking, atrial fibrillation, glucose, total aortic plaque score, heart rate, and brachial-ankle pulse wave velocity*.

### Long-Term Outcomes

**Figure 2:**
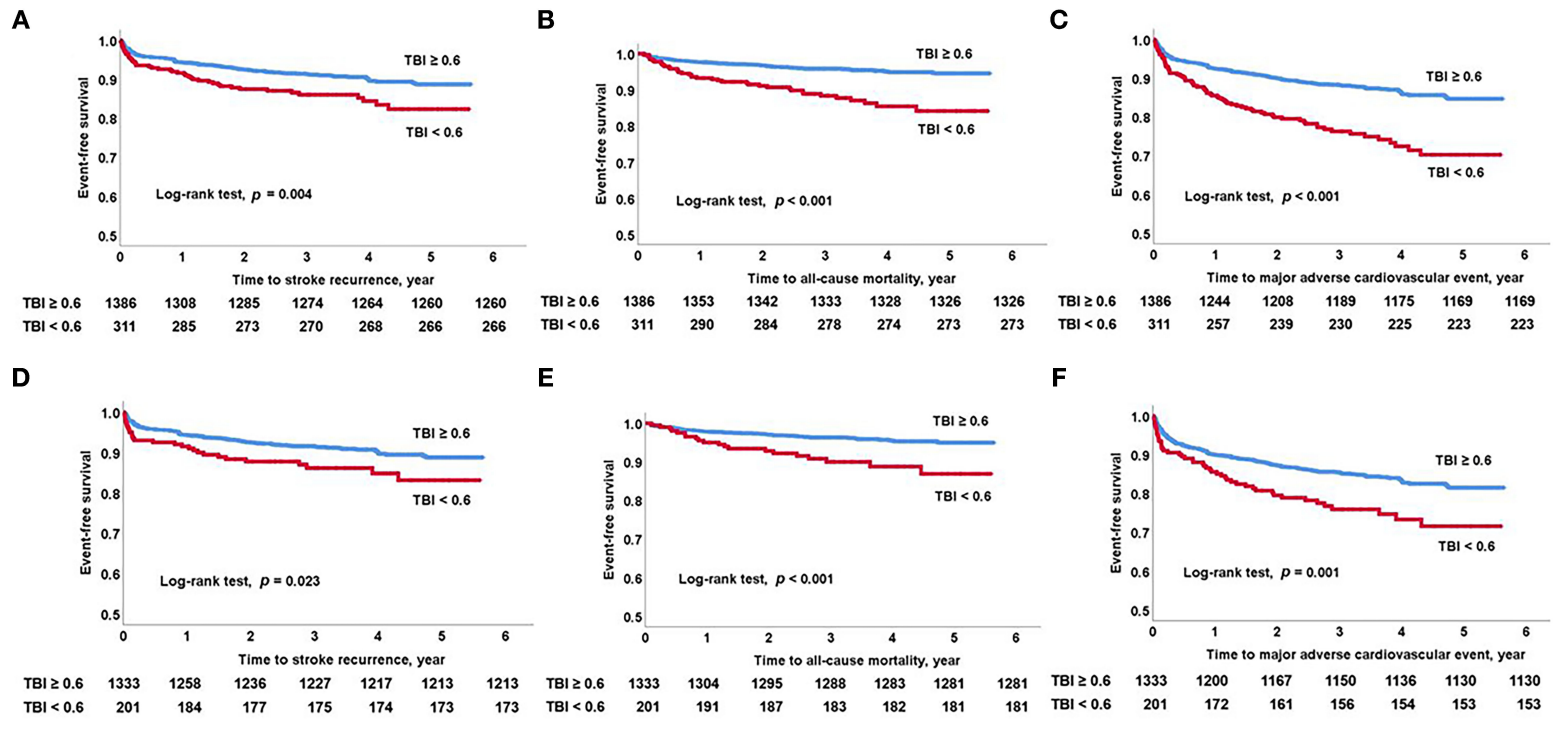
Kaplan–Meier survival analysis. Long-term outcomes as predicted by low TBI. **(A)** Stroke recurrence, **(B)** all-cause mortality, and **(C)** major adverse cardiovascular event in all study patients. **(D)** Stroke recurrence, **(E)** all-cause mortality, and **(F)** major adverse cardiovascular events in patients with normal ABI. ABI, ankle-brachial index; TBI, toe-brachial index.

The study population was followed up for a median of 39.7 (interquartile range, 25.7–54.6) months. A total of 305 patients suffered MACE (18.0%), including 171 stroke recurrences (10.1%), 78 myocardial infarction occurrences (4.6%), and 98 all-cause deaths (5.8%) during the study period. The Kaplan–Meier survival curves showed that the TBI < 0.6 cutoff was significantly associated with high stroke recurrence, all-cause mortality, and MACE in all study patients (log-rank test; all *p* < 0.05). In patients with normal ABI, TBI < 0.6 cutoff was also associated with high stroke recurrence, all-cause mortality, and MACE (log-rank test; all *p* < 0.05) (Figure 2).

To adjust for covariates (age, sex, initial stroke severity, hypertension, diabetes, smoking, atrial fibrillation, glucose, total aortic plaque score, heart rate, and baPWV), multivariable Cox proportional hazards regression analysis was conducted (Table 3). In all study patients, a continuous variable of TBI was independently associated with stroke recurrence [hazard ratio (HR) 0.270, 95% CI 0.090–0.810; *p* = 0.019], all-cause mortality (HR 0.173, 95% CI 0.045–0.671; *p* = 0.011), and MACE (HR 0.254, 95% CI 0.110–0.584; *p* = 0.001). The TBI < 0.6 cutoff was also independently associated with stroke recurrence (HR 1.651, 95% CI 1.135–2.400; *p* = 0.009), all-cause mortality (HR 2.105, 95% CI 1.343–3.298; *p* = 0.001), and MACE (HR 1.838, 95% CI 1.396–2.419; *p* < 0.001). In patients with normal ABI, a continuous variable of TBI was independently associated with all-cause mortality (HR 0.103, 95% CI 0.017–0.618; *p* = 0.013). The TBI < 0.6 cutoff was also independently associated with stroke recurrence (HR 1.681, 95% CI 1.080–2.618; *p* = 0.022), all-cause mortality (HR 2.075, 95% CI 1.180–3.651; *p* = 0.011), and MACE (HR 1.619, 95% CI 1.149–2.281; *p* = 0.006).

**Table 3:** Cox proportional hazards regression analysis of TBI for long-term outcomes.

	**Stroke recurrence^a^**		**All-cause mortality^a^**		**MACE^a^**	
	**HR (95% CI)**	***p*-value**	**HR (95% CI)**	***p-*value**	**HR (95% CI)**	***p-*value**
**All patients (*****n*** **=** **1,697)**						
TBI	0.270 (0.090–0.810)	0.019	0.173 (0.045–0.671)	0.011	0.254 (0.110–0.584)	0.001
TBI <0.6	1.651 (1.135–2.400)	0.009	2.105 (1.343–3.298)	0.001	1.838 (1.396–2.419)	<0.001
**Patients with normal ABI (*****n*** **=** **1,534)**						
TBI	0.281 (0.074–1.067)	0.062	0.103 (0.017–0.618)	0.013	0.379 (0.132–1.087)	0.071
TBI <0.6	1.681 (1.080–2.618)	0.022	2.075 (1.180–3.651)	0.011	1.619 (1.149–2.281)	0.006

*Data were derived from multivariable Cox proportional hazards regression analysis. ABI, ankle-brachial index; CI, confidence interval; HR, hazard ratio; MACE, major adverse cardiovascular event; NIHSS, National Institutes of Health Stroke Scale; TBI, toe-brachial index*.

a *adjusted for age, sex, NIHSS score at admission, hypertension, diabetes mellitus, current smoking, atrial fibrillation, glucose, total aortic plaque score, heart rate, and brachial-ankle pulse wave velocity*.

**Figure 3:**
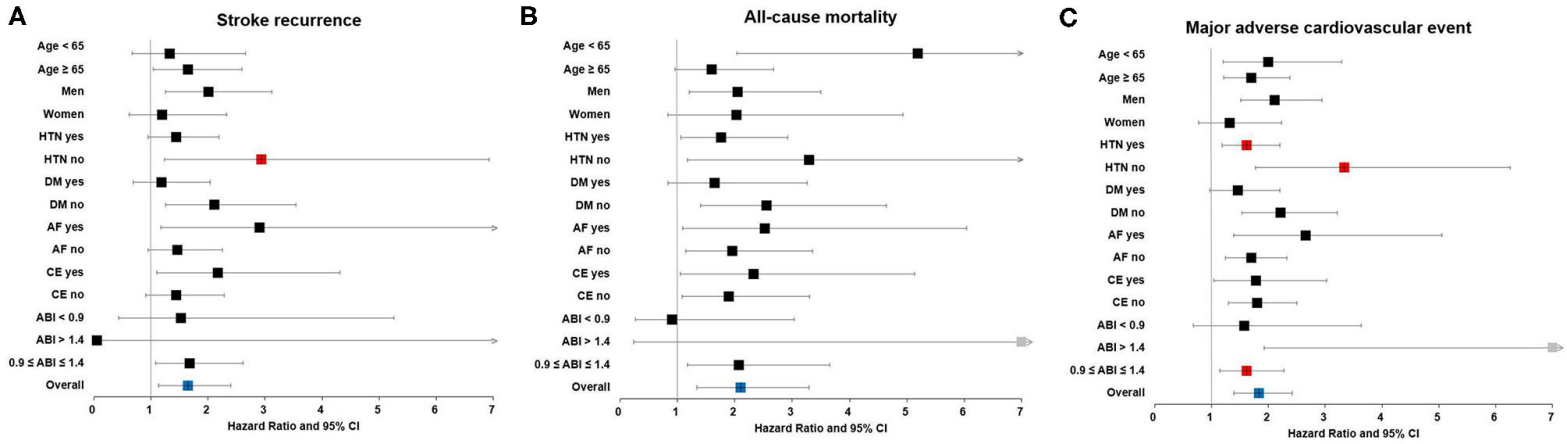
Sensitivity analysis. Association between low TBI and long-term outcomes, including **(A)** stroke recurrence, **(B)** all-cause mortality, and **(C)** major adverse cardiovascular events in patients with acute ischemic stroke. Red square boxes indicate *p* < 0.05 for both interaction and subgroup analysis in multivariable Cox proportional hazards regression. ABI, ankle-brachial index; AF, atrial fibrillation; CE, cardioembolism; CI, confidence interval; DM, diabetes mellitus; HTN, hypertension; TBI, toe-brachial index.

In sensitivity analysis, TBI < 0.6 had significant interactions with hypertension (*p* = 0.040) in stroke recurrence. TBI < 0.6 had significant interactions with hypertension (*p* = 0.006) and ABI status (*p* = 0.049) in MACE. The TBI < 0.6 was associated with stroke recurrence in normotensive patients. The TBI < 0.6 was also associated with MACE regardless of the presence of hypertension. Particularly, TBI < 0.6 was significantly associated with stroke recurrence, all-cause mortality, and MACE in patients with normal ABI (Figure 3 and Supplementary Table 3).

The Harrell's C-index was calculated to compare risk models in survival analysis (Table 4). The baseline model (Model 1) consisted of age, sex, initial stroke severity, hypertension, diabetes, smoking, atrial fibrillation, glucose, total aortic plaque score, heart rate, and baPWV. In all study patients, the C-index for the baseline model was 0.631 (95% CI 0.590–0.672) for stroke recurrence, 0.721 (95% CI 0.674–0.768) for all-cause mortality, and 0.619 (95% CI 0.588–0.650) for MACE. Adding the TBI < 0.6 cutoff (Model 2) improved the prognostic utility of the baseline model in stroke recurrence (C-index 0.647, 95% CI 0.606–0.688), all-cause mortality (C-index 0.744, 95% CI 0.699–0.789), and MACE (C-index 0.640, 95% CI 0.609–0.671). Significant differences were found in all-cause mortality (*p* = 0.037) and MACE (*p* = 0.009). In patients with normal ABI, the C-index for the baseline model was 0.623 (95% CI 0.578–0.668) for stroke recurrence, 0.723 (95% CI 0.664–0.782) for all-cause mortality, and 0.608 (95% CI 0.573–0.643) for MACE. Similarly, adding the TBI < 0.6 cutoff (Model 2) improved the prognostic utility of the baseline model in stroke recurrence (C-index 0.641, 95% CI 0.596–0.686), all-cause mortality (C-index 0.735, 95% CI 0.676–0.794), and MACE (C-index 0.625, 95% CI 0.590–0.660). However, there was no statistically significant difference.

**Table 4:** Harrell's C-index for predicting long-term outcomes.

	**Stroke recurrence**		**All-cause mortality**		**MACE**	
	**C-index (95% CI)**	***p*-value**	**C-index (95% CI)**	***p*-value**	**C-index (95% CI)**	***p*-value**
**All patients (*****n*** **=** **1,697)**						
Model 1^a^	0.631 (0.590 – 0.672)	0.110	0.721 (0.674 – 0.768)	0.037	0.619 (0.588 – 0.650)	0.009
Model 2^b^	0.647 (0.606 – 0.688)		0.744 (0.699 – 0.789)		0.640 (0.609 – 0.671)	
Difference	0.016 (−0.004 – 0.036)		0.023 (0.001 – 0.045)		0.021 (0.005 – 0.037)	
**Patients with normal ABI (*****n*** **=** **1,534)**						
Model 1^a^	0.623 (0.578 – 0.668)	0.134	0.723 (0.664 – 0.782)	0.230	0.608 (0.573 – 0.643)	0.059
Model 2^b^	0.641 (0.596 – 0.686)		0.735 (0.676 – 0.794)		0.625 (0.590 – 0.660)	
Difference	0.018 (−0.006 – 0.042)		0.012 (−0.008 – 0.032)		0.017 (−0.001 – 0.035)	

*Data were derived from Harrell's C-index. ABI, ankle-brachial index; CI, confidence interval; MACE, major adverse cardiovascular event; NIHSS, National Institutes of Health Stroke Scale; TBI, toe-brachial index*.

a *Model 1: age, sex, NIHSS score at admission, hypertension, diabetes mellitus, current smoking, atrial fibrillation, glucose, total aortic plaque score, heart rate, and brachial-ankle pulse wave velocity*.

b *Model 2: Model 1 + TBI < 0.6*.

## Discussion

To the best of our knowledge, this is the first study to investigate the association between TBI and prognosis in patients with acute ischemic stroke. We demonstrated that TBI was inversely and independently associated with poor functional outcome, stroke recurrence, all-cause mortality, and MACE after adjusting for age, cardiovascular risk factors, and central and peripheral atherosclerotic markers. Particularly, low TBI was independently associated with poor short- and long-term outcomes even in patients with normal ABI (0.9 ≤ ABI ≤ 1.4). These findings suggest that low TBI is an independent prognostic indicator for patients with acute ischemic stroke.

The association between PAD and stroke prognosis is well-established (1). ABI has been found a useful non-invasive tool in PAD diagnosis. Patients with PAD (defined as ABI < 0.9) have a 3- to 6-fold increased risk of cardiovascular mortality. Additionally, high ABI (ABI > 1.4) is associated with poor cardiovascular outcome (19). Therefore, both high and low ABIs are independent risk factors, and the prognostic value of ABI has a U-shaped correlation (20). However, falsely elevated ABI may render the ABI less sensitive to the detection of PAD.

The presence of PAD can be diagnosed non-invasively by measuring TBI, as well (21). We found that PAD (defined as TBI < 0.6) was associated with worse stroke outcomes. TBI reflects more peripheral hemodynamics, whereas ABI reflects relatively proximal hemodynamics. In the case of stenotic lesions below the ankle, TBI can be decreased more severely than ABI (22). Several studies have shown that 9–27% patients with suspected PAD have low TBI and normal ABI (9, 23). Our study also showed that 13.1% (201/1,534) of normal ABI patients have low TBI. The prognostic value of low TBI alone has not been reported in patients with acute ischemic stroke. We found that low TBI was more significantly associated with poor prognosis in stroke patients with normal ABI than in those with ABI abnormalities.

Compared with ABI, TBI may be a better marker of atherosclerosis (24). In contrast to ABI, TBI is independent of medial artery calcification and has a linear correlation with PAD severity and poor prognosis (25, 26). In this regard, TBI measurements presumably have more information than ABI for predicting poor prognosis (26). To date, the association between TBI and prognosis has been less frequently studied in elderly populations with diabetes or chronic kidney disease, which are known to have high rates of PAD and medial artery calcification (27–30). Therefore, we conducted additional subgroup analysis in stroke patients with a normal ABI range and found that low TBI was still independently associated with worse outcomes in the subgroup. We also found that the C-index for comparing risk models in survival analysis showed that predicting future death and MACE was improved after adding the TBI < 0.6 cutoff into the baseline model.

Several potential mechanisms for the prognostic ability of low TBI on stroke outcome may be suggested based on our findings. First, we found that low TBI is correlated with several risk factors. Multiple risk factors are significantly associated with stroke outcome (31). Therefore, stroke patients with low TBI are more exposed to risk factors, resulting in a poorer prognosis. Second, we found that atrial fibrillation was 1.5 times more common in patients with low TBI. Atrial fibrillation is a major determinant of stroke recurrence (32) and may exacerbate endothelial damage by abnormal flow shear forces (33). Thus, the coexistence of atrial fibrillation and PAD occurs frequently. The concurrence of atrial fibrillation and PAD may indicate a worse stroke prognosis (34). Despite this possible interaction, multivariate analysis after adjusting for atrial fibrillation showed that low TBI is still an independent predictor of poor outcome. Third, we found that TBI was significantly associated with aortic plaque scores and arterial stiffness measured by baPWV. This means that TBI is closely linked with central and peripheral atherosclerosis. Aortic atheroma indicates a high risk for the occurrence and recurrence of ischemic stroke and poor long-term outcome (35). Arterial stiffness is also an independent prognostic indicator for short- and long-term outcomes in patients with acute ischemic stroke (36–38). Therefore, low TBI may reflect a greater systemic atherosclerotic burden (39).

This study has several noteworthy limitations. First, current guidelines recommend measuring TBI only for individuals with ABI > 1.4. However, both TBI and ABI can be easily measured with the same equipment and take only a few additional minutes. Our study supports the importance of evaluating TBI in addition to ABI in routine clinical practice. Second, we enrolled patients who underwent extensive stroke etiology evaluation, including TEE and continuous electrocardiography monitoring. Our results cannot be adopted to the entire stroke population without extensive evaluation. However, the diagnosis of aortic atheroma and concealed atrial fibrillation makes our findings meaningful. Third, although an increasing number of reports have demonstrated the usefulness of TBI, a grading system for diagnosing PAD needs to be fully established (22, 40). Fourth, TBI and ABI measurements are suggested to be performed twice, but they were performed only once in this study. Fifth, TBI < 0.6 was used in this study instead of TBI < 0.7 suggested by the guidelines. We found that TBI < 0.7 was also an independent long-term prognostic predictor (Supplementary Table 4). Nevertheless, the prognostic effect may differ depending on the cutoff value of TBI. Sixth, atrial fibrillation and heart rate may be the factors that interfere with accurate blood pressure measurement. Thus, we adjusted for atrial fibrillation and heart rate in multivariate analyses. In addition, we conducted further analysis excluding 332 patients (19.6%) with atrial fibrillation or heart rate > 100 bpm. The analysis showed that TBI was still independently associated with poor functional outcome, mortality, and MACE in all patients (Supplementary Tables 5, 6). Lastly, the study population is limited to Korean patients. Our findings need to be confirmed in other populations or cohorts.

## Conclusions

Low TBI is independently associated with poor functional outcome, stroke recurrence, all-cause mortality, and MACE in patients with acute ischemic stroke. These associations remain valid even in stroke patients with normal ABI. Therefore, TBI measurement could be recommended in all patients with ischemic stroke.

## Data Availability Statement

The original contributions presented in the study are included in the article/Supplementary Material, further inquiries can be directed to the corresponding authors.

## Ethics Statement

The studies involving human participants were reviewed and approved by Institutional Review Board of Severance Hospital of Yonsei University Health System. Written informed consent from the participants' legal guardian/next of kin was not required to participate in this study in accordance with the national legislation and the institutional requirements.

## Author Contributions

MH and HN: involved in conceptualization, methodology, providing resources, data curation, project administration, and performed investigation. MH: involved in writing the original draft preparation, performed software analysis, and visualization. YK, IL, HL, and JH: performed data validation. MH and HSL: involved in formal analysis. HN: involved in writing the review and editing, supervision, and funding acquisition. All authors contributed to the article and approved the submitted version.

## Funding

This research was supported by a grant from the Korea Health Technology R&D Project through the Korea Health Industry Development Institute, which is funded by the Ministry of Health & Welfare, Republic of Korea (HI19C0481 and HC19C0028).

## Conflict of Interest

The authors declare that the research was conducted in the absence of any commercial or financial relationships that could be construed as a potential conflict of interest.

## Publisher's Note

All claims expressed in this article are solely those of the authors and do not necessarily represent those of their affiliated organizations, or those of the publisher, the editors and the reviewers. Any product that may be evaluated in this article, or claim that may be made by its manufacturer, is not guaranteed or endorsed by the publisher.
