# Supplementary material for: Low Toe–Brachial Index Is Associated With Stroke Outcome Despite Normal Ankle–Brachial Index
Source: Front Neurol. 2021 Dec 20;12:754258. doi: 10.3389/fneur.2021.754258 (PMC8720783; doi:10.3389/fneur.2021.754258)
Supplement: Supplementary file 1 [file Data_Sheet_1.docx]

Supplementary Material

**Supplementary Table 1. Relationship between risk factors and low TBI.**

|  | TBI ≥ 0.6  (*n* = 1386) | TBI < 0.6  (*n* = 311) | *p* value |
| --- | --- | --- | --- |
|  |  |  |  |
| Hypertension | 1011 (72.9) | 257 (82.6) | <0.001 |
| Diabetes mellitus | 395 (28.5) | 135 (43.4) | <0.001 |
| Hypercholesterolemia | 296 (21.4) | 65 (20.9) | 0.859 |
| Current smoking | 299 (21.6) | 69 (22.2) | 0.812 |
| Coronary artery disease | 471 (34.0) | 114 (36.7) | 0.370 |
| Atrial fibrillation | 226 (16.3) | 80 (25.7) | <0.001 |

Data are expressed as numbers (%). TBI, toe-brachial index.

**Supplementary Table 2. Correlations between vascular markers and TBI.**

|  | TBI | |
| --- | --- | --- |
|  | Pearson’s correlation coefficient | *p* value |
| Complex aortic plaque | -0.220 | <0.001 |
| Simple aortic plaque | -0.184 | <0.001 |
| Total aortic plaque | -0.210 | <0.001 |
| BaPWV, cm/s | -0.115 | <0.001 |
| ABI | 0.530 | <0.001 |

Data were derived from the Pearson’s correlation analysis. ABI, ankle-brachial index; baPWV, brachial-ankle pulse wave velocity; TBI, toe-brachial index.

**Supplementary Table 3. Multivariable Cox regression analysis showing the association between TBI < 0.6 and long-term outcome in subgroup.**

|  | All patients (*n* = 1697) | |  |  |  |  |  |  |  |
| --- | --- | --- | --- | --- | --- | --- | --- | --- | --- |
|  | Stroke recurrence |  |  | All-cause mortality |  |  | MACE |  |  |
|  | Multivariable |  |  | Multivariable |  |  | Multivariable |  |  |
| TBI < 0.6 | HR (95% CI) | *p* value^a^ | *p* value^b^ | HR (95% CI) | *p* value^a^ | *p* value^b^ | HR (95% CI) | *p* value^a^ | *p* value^b^ |
| Overall | 1.651 (1.135‒2.400) | 0.009 |  | 2.105 (1.343‒3.298) | 0.001 |  | 1.838 (1.396‒2.419) | <0.001 |  |
| Age < 65 | 1.331 (0.667‒2.658) | 0.417 | 0.802 | 5.184 (2.036‒13.198) | 0.001 | 0.070 | 1.996 (1.209‒3.295) | 0.007 | 0.541 |
| Age ≥ 65 | 1.645 (1.041‒2.598) | 0.033 |  | 1.602 (0.959‒2.678) | 0.072 |  | 1.703 (1.220‒2.377) | 0.002 |  |
| Men | 2.006 (1.259‒3.199) | 0.003 | 0.621 | 2.053 (1.205‒3.498) | 0.008 | 0.548 | 2.111 (1.512‒2.946) | <0.001 | 0.199 |
| Women | 1.197 (0.617‒2.323) | 0.594 |  | 2.027 (0.834‒4.925) | 0.119 |  | 1.315 (0.774‒2.235) | 0.312 |  |
| HTN yes | 1.443 (0.952‒2.189) | 0.084 | 0.040 | 1.760 (1.060‒2.924) | 0.029 | 0.095 | 1.618 (1.191‒2.200) | 0.002 | 0.006 |
| HTN no | 2.931 (1.241‒6.920) | 0.014 |  | 3.295 (1.173‒9.254) | 0.024 |  | 3.331 (1.775‒6.251) | <0.001 |  |
| DM yes | 1.186 (0.691‒2.037) | 0.536 | 0.061 | 1.648 (0.832‒3.267) | 0.152 | 0.290 | 1.463 (0.972‒2.201) | 0.068 | 0.050 |
| DM no | 2.109 (1.256‒3.541) | 0.005 |  | 2.549 (1.402‒4.636) | 0.002 |  | 2.219 (1.532‒3.213) | <0.001 |  |
| AF yes | 2.904 (1.179‒7.155) | 0.020 | 0.088 | 2.527 (1.095‒6.041) | 0.030 | 0.715 | 2.656 (1.396‒5.054) | 0.003 | 0.165 |
| AF no | 1.466 (0.954‒2.254) | 0.081 |  | 1.959 (1.144‒3.353) | 0.014 |  | 1.704 (1.247‒2.329) | 0.001 |  |
| CE yes | 2.178 (1.100‒4.315) | 0.026 | 0.182 | 2.327 (1.054‒5.138) | 0.037 | 0.728 | 1.780 (1.046‒3.031) | 0.034 | 0.855 |
| CE no | 1.444 (0.912‒2.287) | 0.117 |  | 1.894 (1.087‒3.302) | 0.024 |  | 1.804 (1.299‒2.506) | <0.001 |  |
| ABI < 0.9 | 1.521 (0.440‒5.261) | 0.508 | 0.526 | 0.908 (0.272‒3.039) | 0.876 | 0.430 | 1.573 (0.681‒3.634) | 0.289 | 0.049 |
| ABI > 1.4 | 0.057 (0.000‒4.490e+89) | 0.979 | 0.936 | 2.008e+10 (0.236‒1.711e+21) | 0.065 | NA | 312.684 (1.928‒50720.719) | 0.027 | 0.906 |
| 0.9 ≤ ABI ≤ 1.4 | 1.681 (1.080‒2.618) | 0.022 | ref | 2.075 (1.180‒3.651) | 0.011 | ref | 1.619 (1.149‒2.281) | 0.006 | ref |

Data were derived from multivariable Cox proportional hazards regression analysis. ABI, ankle-brachial index; AF, atrial fibrillation; CE, cardioembolism; CI, confidence interval; DM, diabetes mellitus; HR, hazard ratio; HTN, hypertension; MACE, major adverse cardiovascular event; NIHSS, National Institutes of Health Stroke Scale; TBI, toe-brachial index. ^a^ *p* value adjusted for age, sex, NIHSS score at admission, hypertension, diabetes mellitus, current smoking, atrial fibrillation, glucose, total aortic plaque score, heart rate, and brachial-ankle pulse wave velocity. ^b^ *p* value for interaction between TBI < 0.6 and indicated variable.

**Supplementary Table 4. Cox proportional hazards regression analysis of TBI for long-term outcomes.**

|  | All patients (*n* = 1697) | | |  |  |  |
| --- | --- | --- | --- | --- | --- | --- |
|  | Stroke recurrence |  | All-cause mortality |  | MACE |  |
|  | Multivariable^a^ |  | Multivariable^a^ |  | Multivariable^a^ |  |
|  | HR (95% CI) | *p* value | HR (95% CI) | *p* value | HR (95% CI) | *p* value |
| TBI | 0.270 (0.090‒0.810) | 0.019 | 0.173 (0.045‒0.671) | 0.011 | 0.254 (0.110‒0.584) | 0.001 |
| TBI < 0.7 | 1.584 (1.133‒2.216) | 0.007 | 1.856 (1.188‒2.901) | 0.007 | 1.428 (1.110‒1.836) | 0.006 |
|  | Patients with normal ABI (*n* = 1534) | | |  |  |  |
|  | Stroke recurrence |  | All-cause mortality |  | MACE |  |
|  | Multivariable^a^ |  | Multivariable^a^ |  | Multivariable^a^ |  |
|  | HR (95% CI) | *p* value | HR (95% CI) | *p* value | HR (95% CI) | *p* value |
| TBI | 0.281 (0.074‒1.067) | 0.062 | 0.103 (0.017‒0.618) | 0.013 | 0.379 (0.132‒1.087) | 0.071 |
| TBI < 0.7 | 1.482 (1.033‒2.125) | 0.033 | 1.832 (1.100‒3.052) | 0.020 | 1.307 (0.988‒1.728) | 0.060 |

Data were derived from multivariable Cox proportional hazards regression analysis. ABI, ankle-brachial index; CI, confidence interval; HR, hazard ratio; MACE, major adverse cardiovascular event; NIHSS, National Institutes of Health Stroke Scale; TBI, toe-brachial index. ^a^ adjusted for age, sex, NIHSS score at admission, hypertension, diabetes mellitus, current smoking, atrial fibrillation, glucose, total aortic plaque score, heart rate, and brachial-ankle pulse wave velocity.

**Supplementary Table 5. Logistic regression analysis of TBI for poor functional outcomes at 3 months in patients without atrial fibrillation or heart rate > 100 bpm.**

|  | All patients (*n* = 1365) |  |  |  |
| --- | --- | --- | --- | --- |
|  | Univariable |  | Multivariable^a^ |  |
|  | OR (95% CI) | *p* value | OR (95% CI) | *p* value |
| TBI | 0.285 (NA) | <0.001 | 0.263 (0.087‒0.794) | 0.018 |
| TBI < 0.6 | 0.383 (NA) | <0.001 | 1.419 (0.963‒2.090) | 0.077 |
|  | Patients with normal ABI (*n* = 1246) | |  |  |
|  | Univariable |  | Multivariable^a^ |  |
|  | OR (95% CI) | *p* value | OR (95% CI) | *p* value |
| TBI | 0.365 (NA) | <0.001 | 0.313 (0.082‒1.187) | 0.088 |
| TBI < 0.6 | 0.365 (NA) | <0.001 | 1.319 (0.822‒2.116) | 0.251 |

Data were derived from multivariable logistic regression analysis. ABI, ankle-brachial index; CI, confidence interval; NIHSS, National Institutes of Health Stroke Scale; OR, odds ratio; TBI, toe-brachial index. ^a^ adjusted for age, sex, NIHSS score at admission, hypertension, diabetes mellitus, current smoking, glucose, total aortic plaque score, and brachial-ankle pulse wave velocity.

**Supplementary Table 6. Cox proportional hazards regression analysis of TBI for long-term outcomes in patients without atrial fibrillation or heart rate > 100 bpm.**

|  | All patients (*n* = 1365) | | |  |  |  |
| --- | --- | --- | --- | --- | --- | --- |
|  | Stroke recurrence |  | All-cause mortality |  | MACE |  |
|  | Multivariable^a^ |  | Multivariable^a^ |  | Multivariable^a^ |  |
|  | HR (95% CI) | *p* value | HR (95% CI) | *p* value | HR (95% CI) | *p* value |
| TBI | 0.379 (0.112‒1.284) | 0.119 | 0.100 (0.020‒0.493) | 0.005 | 0.261 (0.103‒0.662) | 0.005 |
| TBI < 0.6 | 1.420 (0.920‒2.192) | 0.113 | 2.212 (1.279‒3.824) | 0.004 | 1.737 (1.268‒2.378) | 0.001 |
|  | Patients with normal ABI (*n* = 1246) | | |  |  |  |
|  | Stroke recurrence |  | All-cause mortality |  | MACE |  |
|  | Multivariable^a^ |  | Multivariable^a^ |  | Multivariable^a^ |  |
|  | HR (95% CI) | *p* value | HR (95% CI) | *p* value | HR (95% CI) | *p* value |
| TBI | 0.475 (0.105‒2.157) | 0.335 | 0.149 (0.015‒1.450) | 0.013 | 0.602 (0.180‒2.013) | 0.410 |
| TBI < 0.6 | 1.331 (0.772‒2.294) | 0.304 | 1.532 (0.709‒3.309) | 0.278 | 1.327 (0.870‒2.024) | 0.189 |

Data were derived from multivariable Cox proportional hazards regression analysis. ABI, ankle-brachial index; CI, confidence interval; HR, hazard ratio; MACE, major adverse cardiovascular event; NIHSS, National Institutes of Health Stroke Scale; TBI, toe-brachial index. ^a^ adjusted for age, sex, NIHSS score at admission, hypertension, diabetes mellitus, current smoking, glucose, total aortic plaque score, and brachial-ankle pulse wave velocity.
